# Saberes profanos y justicia epistémica en salud mental: análisis crítico del agente de apoyo entre iguales en el Estado español

**DOI:** 10.18294/sc.2026.6041

**Published:** 2026-02-26

**Authors:** Diana Gonzalez-Mañas, Martín Correa-Urquiza

**Affiliations:** 1 Autora de correspondencia. Máster en Antropología Médica y Salud Global. Técnica de Investigación, Universidad de Barcelona. Barcelona, España. dianagonzalez@ub.edu Universitat de Barcelona Técnica de Investigación Universidad de Barcelona Barcelona Spain dianagonzalez@ub.edu; 2 Doctor en Antropología Médica y Salud Internacional. Profesor Serra Hunter, Departamento de Antropología, Filosofía y Trabajo Social. Universidad Rovira i Virgili. Tarragona. España. martin.correaurquizav@urv.cat Universitat Rovira i Virgilu Departamento de Antropología Filosofía y Trabajo Social Universidad Rovira i Virgili Tarragona Spain martin.correaurquizav@urv.cat

**Keywords:** Salud Mental, Epistemología, Antropología Médica, Derechos Humanos, España

## Abstract

El presente artículo, de carácter ensayístico, examina críticamente la figura del agente de apoyo entre iguales en el sistema de salud mental del Estado español, como dispositivo situado en la tensión entre la transformación de las jerarquías epistémicas y el riesgo de cooptación institucional. Partiendo de nuestras experiencias e investigaciones en el campo de la antropología médica crítica y los derechos humanos en salud mental, junto y desde los activismos del Movimiento Loco en el Estado español, analizamos cómo esta figura profesional encarna simultáneamente una promesa de redistribución del poder epistémico y el peligro de neutralización por parte del modelo biomédico hegemónico. Sostenemos que la justicia epistémica en salud mental requiere la transformación radical de las condiciones materiales, relacionales e institucionales que han sostenido históricamente la deslegitimación de los saberes experienciales. A través de un análisis genealógico del agente de apoyo entre iguales y de sus procesos de institucionalización, identificamos las condiciones estructurales que determinan si su incorporación puede efectivamente cuestionar los regímenes de verdad dominantes o si, por el contrario, opera como mecanismo de legitimación que incorpora formalmente la diversidad sin transformar las lógicas de poder existentes. Concluimos proponiendo un marco de cuatro dimensiones de intervención política: el fortalecimiento de las fraternidades epistémicas y las redes del Movimiento Loco como activos comunitarios, la transformación de las culturas profesionales, la disputa de marcos regulativos y la articulación de procesos de coproducción local de conocimiento.

## Introducción

Históricamente, el ámbito de la salud mental ha sido un campo de profundas desigualdades epistémicas. Si bien el campo psiquiátrico ha estado atravesado por disputas internas, corrientes críticas y experiencias contrahegemónicas^1^^,^^2^^,^^3^, la orientación biomédica dominante ha tendido a establecer relaciones asimétricas con los saberes derivados de la experiencia vivida de las personas psiquiatrizadas. Desde el nacimiento de la psiquiatría moderna, aunque han existido posturas e iniciativas que cuestionaron el reduccionismo biomédico y el silenciamiento de la experiencia vivida^4^^,^^5^^,^^6^, estas corrientes han ocupado posiciones más bien intersticiales dentro del poder psiquiátrico, sin lograr desplazar la hegemonía del paradigma biomédico en las instituciones, las políticas públicas o la formación profesional, y privilegiando marcos interpretativos centrados en el diagnóstico, la patologización y la intervención por encima del reconocimiento de las personas psiquiatrizadas como sujetos productores de conocimiento válido^7^^,^^8^^,^^9^^,^^10^. En virtud de este ordenamiento epistemológico, las personas psiquiatrizadas han sido configuradas como sujetos subalternos privados de agencia epistémica^11^ y despojados de la palabra, en términos de Huertas^12^. A la luz de Miranda Fricker^13^, esta deslegitimación se conoce como injusticia epistémica: una forma distintiva de agravio que afecta a los sujetos en su condición de conocedores y se manifiesta paradigmáticamente allí donde determinados sujetos o colectivos son sistemáticamente desacreditados como portadores de conocimiento válido debido a prejuicios estructurales que operan sobre su identidad social. 

En el contexto específico de las personas psiquiatrizadas, esta injusticia se expresa tanto en su vertiente testimonial (cuando su palabra es recibida con un déficit de credibilidad, o directamente invalidada), como en su manifestación hermenéutica (cuando carecen de los recursos interpretativos necesarios para articular y hacer inteligible su experiencia en un contexto sociocultural que ha marginado sus voces de los procesos de construcción del lenguaje legitimado)^11^^,^^13^^,^^14^. No obstante, en las últimas décadas se ha producido un giro epistemológico que cuestiona esta distribución jerárquica del saber. La emergencia de lo que ha sido conceptualizado bajo la denominación de saberes profanos^10^ ha irrumpido como una respuesta política y epistémica al silenciamiento histórico y ha configurado un horizonte que busca reparar las formas de injusticia epistémica que, siguiendo a Fricker, han perjudicado sistemáticamente a las personas psiquiatrizadas, tanto en su condición de conocedoras como en su capacidad para articular e interpretar su propia experiencia. 

En el contexto específico del Estado español, la progresiva expansión y consolidación del Movimiento Loco o en primera persona ha evidenciado la necesidad de reconsiderar la distribución asimétrica de legitimidad en el campo de la producción epistémica^15^. Estas iniciativas han problematizado de manera explícita y contundente el hecho de que, durante siglos, quienes han experimentado directamente la locura hayan sido estructuralmente excluidos de los espacios institucionales de producción discursiva^16^. En este marco interpretativo, la enunciación “en primera persona” es una de las formas de conceptualizar las dimensiones singulares y plurales de individuos, asociaciones, colectivos y entidades desarrolladas por personas con sufrimiento psíquico que han vivido experiencias de psiquiatrización. No es una categoría cerrada ni ajena a tensiones, pero actualmente se utiliza para hablar de aquello que emerge desde los propios individuos y colectivos. En definitiva, ha constituido el acto político de abandonar la condición de objeto pasivo del discurso ajeno para reclamar la potestad legítima de interpretar, de nominar y de construir alternativas paradigmáticas^15^^,^^17^^,^^18^^,^^19^. 

Los saberes profanos constituyen, en este contexto, un cuerpo de conocimiento que emana directamente de la experiencia vivencial de las aflicciones, pero no solamente acerca del sufrimiento, sino que constituyen el propio fundamento desde el cual se configura el sentido de la experiencia vivida y se articulan las posibles estrategias para un mayor bienestar. Representa, en última instancia, una praxis transformadora orientada a generar condiciones materiales y simbólicas para un diálogo horizontal en el que las personas psiquiatrizadas sean finalmente reconocidas como sujetos productores de sentido y no meramente como objetos de intervención o conocimiento.

La emergencia de los saberes profanos, sin embargo, no se produce en un terreno epistemológicamente neutral. Desde la perspectiva crítica de la antropología médica, Arthur Kleinman^20^ analiza la distinción entre los diversos marcos epistemológicos en el campo de la salud y establece una diferenciación teórica entre los conceptos de “*disease*”, “*illness*” y “*sickness*”. Para Kleinman, la experiencia subjetiva del sufrimiento (*illness*) y las formas socioculturales de interpretarlo (*sickness*) quedan frecuentemente invisibilizadas por la narrativa biomédica hegemónica, centrada exclusivamente en la conceptualización objetivada de la enfermedad (*disease*). Siguiendo esta línea teórica, Martínez-Hernáez y Correa-Urquiza^15^ proponen conceptualizar los saberes profanos como saberes nómadas que se posicionan lateralmente respecto al dispositivo experto institucionalizado. Estos conocimientos no se originan tradicionalmente en una disciplina académica formalizada ni se articulan desde una lógica de autoridad, sino que emergen en el intersticio liminal entre la experiencia encarnada y la necesidad existencial. Su naturaleza esencialmente nómada los hace resistentes a la codificación institucional y, precisamente por esta característica, potencialmente disruptivos respecto al orden epistémico establecido. Por tanto, lo que está fundamentalmente en juego en el campo de los saberes profanos no es simplemente la posibilidad de reconocer formalmente a un sujeto otro, sino de transformar radicalmente las condiciones estructurales de producción del conocimiento en el campo de la salud mental y desarticular el relato hegemónico que ha definido históricamente lo vivido desde una exterioridad objetivante. 

Partiendo de nuestras experiencias e investigaciones en el campo de la antropología médica y los derechos humanos en salud mental, junto y desde los activismos del Movimiento Loco en el Estado español, este artículo examina críticamente las posibilidades, tensiones y condiciones en las que el agente de apoyo entre iguales puede contribuir a una redistribución efectiva de la autoridad y legitimidad epistémica en el sistema de salud mental, y en qué escenarios corre el riesgo de ser absorbido, neutralizado o instrumentalizado por las lógicas del sistema experto. 

En este sentido, cuando hablamos de sistema de salud mental nos referimos al entramado de dimensiones culturales, económicas e institucionales que interviene desde los dispositivos asistenciales hasta los marcos regulativos, los modos de financiación, el diseño de políticas públicas, los protocolos de intervención y las culturas profesionales que atraviesan dichos dispositivos y recursos. Reconociendo la diversidad de orientaciones epistemológicas, paradigmas de intervención e iniciativas que promueven la transformación de los sistemas de salud mental hacia modelos basados en derechos humanos^21^^,^^22^^,^^23^, su heterogeneidad no impide que el saber biomédico-psiquiátrico haya ocupado una posición hegemónica en la definición de lo patológico y, en consecuencia, una posición hegemónica en la definición de la recuperación^24^. Es precisamente sobre esta tensión donde el agente de apoyo entre iguales puede evidenciar las condiciones de posibilidad para una transformación radical.

El ensayo se estructura en torno a tres ejes. En primer lugar, examinaremos la figura del agente de apoyo entre iguales como dispositivo situado entre saberes, atendiendo a su genealogía y a sus potencialidades para reconfigurar las relaciones epistémicas y de poder. En segundo lugar, analizaremos críticamente los riesgos de cooptación institucional que pueden neutralizar su capacidad transformadora cuando el agente de apoyo entre iguales se integra bajo lógicas profesionales que reproducen las jerarquías que originalmente cuestionaba. Finalmente, propondremos un marco de condiciones estructurales necesarias para avanzar hacia la justicia epistémica en salud mental, entendida no como un estado alcanzable mediante la incorporación formal del agente de apoyo entre iguales, sino como un horizonte político que exige transformar radicalmente las condiciones de producción y legitimación del conocimiento. A lo largo de este recorrido, el agente de apoyo entre iguales no se presenta como una solución en sí misma a la transformación del sistema de salud mental, sino como un analizador privilegiado que permite interrogar sus condiciones de posibilidad y sus límites estructurales. En última instancia, el agente de apoyo entre iguales no parece resolver naturalmente el problema de la injusticia epistémica, pero lo hace visible y lo instala como cuestión política ineludible.

## Genealogía y potencialidades del agente de apoyo entre iguales

La figura profesional del agente de apoyo entre iguales (del inglés *peer support worker*) constituye una innovación crítica en el campo de la salud mental que responde, en gran medida, a los complejos procesos de politización del sufrimiento psíquico y de legitimación de los saberes profanos que han experimentado una notable expansión durante las últimas décadas^25^^,^^26^. Aunque se documentan experiencias primigenias de apoyo entre iguales en salud mental ya en el siglo XVIII^27^, sus raíces toman fuerza en los movimientos de usuarios y supervivientes de la psiquiatría que emergieron en las décadas de 1960 y 1970, particularmente en EEUU y Europa Occidental^28^^,^^29^. Estos movimientos surgieron como respuesta crítica a las prácticas de institucionalización, las prácticas coercitivas y la exclusión sistemática de las personas psiquiatrizadas de los espacios de decisión sobre sus propias vidas^30^. Organizaciones pioneras como el *Mental Patients’ Liberation Front* en Boston o la *Campaign Against Psychiatric Oppression* en el Reino Unido articularon formas de apoyo horizontal que cuestionaban el monopolio profesional sobre el cuidado y reivindicaban la experiencia vivida como fuente legítima de conocimiento y autoridad^31^. En este contexto, a principios de la década de 1980, Gartner y Riessman^32^ describieron el apoyo mutuo en salud mental como un proceso de ayuda entre personas que comparten experiencias o dificultades similares, orientado tanto al cambio personal como a la transformación social. Posteriormente, ya en el inicio del siglo XXI, Mead, Hilton y Curtis^33^ ampliaron esta conceptualización al entender el apoyo entre iguales como una relación recíproca de intercambio sustentada en el respeto, la corresponsabilidad y la construcción conjunta de aquello que resulta útil para quienes participan en dicha relación. En suma, el apoyo entre iguales se concibe como una práctica política que resiste a las relaciones de poder inherentes al saber biomédico-psiquiátrico y promueve formas de mutualidad basadas en la comprensión compartida del sufrimiento^33^^,^^34^^,^^35^. 

A partir de los años 1990, a razón de la aparición del modelo de recuperación^36^, el apoyo entre iguales comenzó a formalizarse e institucionalizarse progresivamente, integrándose en diversos grados dentro de los sistemas de salud mental de numerosos países^33^^,^^37^^,^^38^ y generando debates y controversias sobre la mejor manera de integrar en los servicios una fuerza originalmente concebida como alternativa y crítica^39^^,^^40^^,^^41^^,^^42^^,^^43^. El agente de apoyo entre iguales encarna, al menos en su formulación epistemológica y ontológica ideal, una tentativa radical de fracturar el tradicional monopolio del saber experto y de reconocer la experiencia vivida como fuente legítima de conocimiento, promoviendo la configuración de modalidades innovadoras de acompañamiento caracterizadas por la horizontalidad y una mayor permeabilidad a la singularidad irreductible de las trayectorias vitales subjetivas^28^^,^^44^^,^^45^. Su posicionamiento epistémico diferencial radica en que construye conocimiento desde la experiencia encarnada, implementa prácticas de escucha desde la inmediatez existencial del “haber estado ahí” y desarrolla modalidades de acompañamiento desde la proximidad vivencial^46^^,^^47^. En determinados contextos institucionales, el agente de apoyo entre iguales se desempeña fundamentalmente como acompañante en procesos de recuperación personal y, en otros, como facilitador de grupos de apoyo mutuo o como mediador entre las personas usuarias y los profesionales sanitarios^33^^,^^48^. En el Estado español, la incorporación del agente de apoyo entre iguales se ha implementado de forma desigual y todavía incipiente, con avances significativos pero fragmentados entre comunidades autónomas^26^. La mayoría de las iniciativas surgen de asociaciones y entidades del tercer sector, mientras que la incorporación al sistema público es limitada y sin una categoría profesional homogénea. 

Entre las potencialidades más significativas de esta figura intersticial se encuentra su capacidad para configurar y sostener relaciones de cuidado horizontal, fundamentadas en prácticas de escucha encarnada, empatía situada y reconocimiento mutuo^49^. Uno de los aspectos más valorados por las personas que son acompañadas por agentes de apoyo entre iguales es precisamente la posibilidad de construir una identidad no reducida a la condición patológica, de recuperar la agencia sobre la propia narrativa vital y de visualizar futuros alternativos al determinismo del pronóstico psiquiátrico^50^. Además, esta figura puede contribuir potencialmente a redistribuir las relaciones de poder instituidas dentro de los dispositivos asistenciales. De manera paradigmática, el proceso de desinstitucionalización en Trieste (Italia) fue de la mano de la incorporación de personas psiquiatrizadas como trabajadoras de los servicios comunitarios de la región italiana que, a su vez, se desarrolló simultáneamente con un cuestionamiento radical de las lógicas manicomiales, una democratización efectiva de las relaciones de cuidado y una politización explícita de las prácticas institucionales^51^^,^^52^. En este contexto, la articulación entre transformación institucional y reconocimiento de los saberes profanos demostró su efectividad en términos tanto clínicos como sociales, lo que convirtió el modelo triestino en un referente internacional de democratización del campo de la salud mental.

## Deriva del par y neutralización institucional

No obstante, sus potencialidades transformadoras no deben conducirnos a una concepción idealizada o acrítica del agente de apoyo entre iguales, ni a ignorar los múltiples riesgos estructurales que rodean su proceso de institucionalización. Uno de los principales peligros identificados por la literatura aparece cuando su potencial transformador coexiste dialécticamente con el riesgo permanente de neutralización institucional y cooptación sistémica^53^, convirtiéndose progresivamente en una versión institucionalmente blanqueada del buen paciente, en lugar de preservar su potencialidad como figura crítica, disruptiva y colectivamente articulada. Cuando al agente de apoyo entre iguales se le asignan funciones instrumentales sin reconocimiento epistémico efectivo, se le exige reproducir acríticamente las lógicas preexistentes o sus responsabilidades comienzan a desviarse de las prácticas distintivas que los diferencian del resto de profesionales, su capacidad disruptiva se puede ver severamente limitada^54^. A este fenómeno se lo conoce como “deriva del par”, del inglés *peer drift*, y es considerado uno de los principales retos para su integración en los servicios de salud mental^55^^,^^56^. En particular, surge la cuestión de si el apoyo entre iguales mantiene su esencia original cuando se traslada desde entornos cotidianos y comunitarios al contexto estructurado y regulado y de los sistemas de salud mental; si, al institucionalizarse, sigue siendo el mismo tipo de apoyo en términos de autenticidad, o si su significado y función se transforman dentro de estos sistemas^57^. 

Una manifestación paradigmática de esta deriva neutralizadora es la figura institucional del “paciente experto”^10^, que no es una iniciativa de apoyo entre iguales como tal, pero que también parte del hecho de haber vivido una experiencia concreta de salud mental. En estos casos, el sistema sanitario reconoce formalmente la voz de la experiencia exclusivamente cuando esta se subordina a sus marcos interpretativos preestablecidos: cuando el sujeto designado como “experto” reproduce acríticamente el discurso biomédico hegemónico, promueve activamente la psicoeducación y la adherencia al tratamiento farmacológico o desempeña funciones de intermediación entre los profesionales sanitarios y las personas usuarias. La evidencia también documenta que algunas personas que han asumido este rol institucional terminan experimentando sentimientos de aislamiento, desprotección o instrumentalización por parte de estructuras organizativas que no reconocen adecuadamente su especificidad epistémica ni su aportación diferencial^58^^,^^59^^,^^60^. Desde los sistemas de atención, se han identificado obstáculos estructurales como, por ejemplo, dificultades para la integración efectiva en equipos multidisciplinares, persistencia de actitudes escépticas o abiertamente hostiles por parte de profesionales formados en modelos biomédicos tradicionales, ambigüedades y conflictos en la delimitación de roles y responsabilidades, ausencia de protocolos claros sobre confidencialidad y límites éticos, así como culturas organizativas rígidas que dificultan la adaptación a formas de trabajo basadas en la horizontalidad y la mutualidad^61^^,^^62^

En suma, el carácter híbrido del agente de apoyo entre iguales lo coloca ante el riesgo permanente de que estos saberes sean instrumentalizados por las instituciones hegemónicas y reconocidos selectivamente solo en la medida en que se subordinan a la lógica biomédica dominante. Sin embargo, las críticas más profundas provienen precisamente de los movimientos en primera persona, que problematizan la profesionalización misma del apoyo entre iguales como potencial traición a sus fundamentos emancipatorios. Su preocupación radica en la transformación del agente de apoyo entre iguales en “experto” o poseedor de un “saber técnico” que, paradójicamente, podría reproducir las mismas jerarquías epistémicas y relacionales que el movimiento originalmente cuestionaba. En ese caso, en lugar de producirse una transformación del sistema, es la propia persona quien termina adaptándose a sus normas, valores y formas de funcionamiento^26^. 

También se advierte que un anclaje excesivo en la identidad de sujeto usuario de servicios puede dificultar la integración profesional y limitar la capacidad de incidencia dentro de los equipos^26^. Así, en lugar de habilitar espacios de reconocimiento epistémico efectivo, la incorporación del agente de apoyo entre iguales en el sistema de salud mental puede clausurar el conflicto inherente a la diversidad de saberes, reducir instrumentalmente el saber profano a una función técnica subordinada y neutralizar su capacidad transformadora^63^. Parte de lo expuesto puede vincularse a lo que la filósofa india Gayatri Chakravorty Spivak denuncia cuando afirma que no es que el subalterno (entendido como posición, no como identidad) no esté en condiciones de hablar, sino que las posibilidades de escucha y resonancia de su palabra están socialmente limitadas^64^. Para Spivak, el subalterno fracasa en su intento de comunicarse porque su mensaje queda encapsulado en un modo interpretativo que individualiza la enunciación vinculándola a problemas personales, en este caso, supuestamente ligados al sufrimiento psíquico. En otras palabras, no se trataría de una condición de ausencia de voz o mutismo, sino de las interpretaciones cerradas que se generan de su acto o mensaje y que impiden que la información sea acogida y reconocida. 

A esto habría de añadirse la necesidad de pensar si más allá de las formaciones en apoyo entre iguales existentes^26^^,^^65^^,^^66^, esta figura no debería ser resultado también de procesos de construcción compartida de significaciones, de un cuerpo-texto variado y mínimamente consensuado que surja de los movimientos sociales organizados. Fernando Broncano^67^ afirma que la experiencia vivida de la opresión y subalternización no basta, por sí sola, para desarrollar una conciencia crítica sobre la situación; hace falta el encuentro y la problematización con un otro semejante. Para Broncano, el saber profano no se agota en la experiencia individual; requiere de comunidades epistémicas donde lo vivido pueda articularse como conocimiento colectivo y político. En este sentido, el papel del agente de apoyo entre iguales como fraternidad epistémica^18^^,^^67^ puede ser fundamental para una integración que refleje el poder emancipatorio de los movimientos en los que el apoyo entre iguales tiene su origen. Por ello, resulta igualmente esencial que este agente mantenga y desarrolle lazos de conexión con los movimientos sociales vinculados al Movimiento Loco, en tanto constituyen territorios privilegiados de producción de conciencia crítica y de generación de conocimiento epistémico colectivo. Si vamos a incorporar a agentes de apoyo entre iguales en el sistema de salud mental, tal vez deberíamos reconocer que representan una forma de conocimiento colectivo construido en comunidad (fraternidad epistémica) cuya inclusión en los equipos asistenciales debe mantener vivo el espíritu transformador y liberador de los movimientos sociales de los que surgieron. 

En cuanto al saber profano, quizás sea reduccionista definirlo o circunscribirlo a aquello que deriva de la experiencia. Sin duda es, al mismo tiempo, un saber que no se acota o no es solamente desde la experiencia, sino que es un saber sobre o en relación con la dimensión existencial del sufrimiento, una dimensión frecuentemente olvidada por las disciplinas basadas en síntomas. El saber profano no es solo una historia personal. Es también una forma de sabiduría sobre la condición humana, sobre cómo el sufrimiento existencial se manifiesta y se vive. Este conocimiento filosófico-existencial, que las personas con experiencias de psiquiatrización pueden tener, complementa y enriquece el modelo biomédico centrado en síntomas. 

El psiquiatra holandés Jim Van Os, en una entrevista reciente^68^, recuerda que hay una dimensión existencial en la aflicción psíquica que ha sido sistemáticamente descartada por el modelo actual de atención. El reduccionismo frecuente en el campo de la salud mental nos ha llevado a la falacia de creer que estos sufrimientos son solo realidades patológicas que se manifiestan en forma de síntomas y que los diagnósticos son realidades naturales que pesan sobre el cerebro de los individuos. La dimensión existencial, frecuentemente eclipsada por los lenguajes sintomatológicos, constituye precisamente uno de los núcleos epistémicos desde los cuales emergen los saberes profanos y abre la posibilidad de entender los malestares de una forma más compleja en la que no es posible reducir la experiencia vivida a un conjunto de síntomas. 

## De la incorporación formal a la transformación estructural: condiciones para la justicia epistémica

A la luz de Fricker, la justicia epistémica no parece ser un estado que pueda alcanzarse de forma definitiva, sino un horizonte ético y político que exige el reconocimiento de otras formas de saber, el desmantelamiento de las jerarquías epistémicas y la creación de contextos institucionales y comunitarios que habiliten el diálogo genuino entre experiencias, disciplinas y modos de comprender el sufrimiento^69^. En ese sentido, el agente de apoyo entre iguales puede entenderse como una figura-límite, situada en el umbral entre saberes históricamente jerarquizados: puede ser a la vez una promesa y un riesgo, un puente y una tensión, una oportunidad para reconfigurar las relaciones entre saberes, siempre que su potencial crítico no se vea neutralizado por las lógicas de la profesionalización, al servicio del sistema experto. Dado que la figura del agente de apoyo entre iguales se ha revelado como una posición liminal^70^, su emergencia puede comprenderse tanto como síntoma de una apertura de los saberes expertos a los saberes subalternos como también, paradójicamente, un intento de contener y redirigir dicha emergencia hacia lógicas funcionales a los marcos existentes. De ahí la necesidad de desplazar la reflexión desde valoraciones abstractas sobre el potencial de esta figura hacia interrogaciones concretas sobre su configuración práctica en contextos institucionales específicos. ¿Qué tipo de tensiones atraviesan efectivamente el ejercicio cotidiano del agente de apoyo entre iguales cuando sus saberes y posicionamientos entran en contacto con los dispositivos clínicos? ¿Se configuran verdaderos espacios de deliberación y construcción horizontal? ¿Hasta qué punto puede esta figura facilitar un desplazamiento del saber experto hacia formas de conocimiento encarnadas? ¿Está ampliando los márgenes de lo decible en salud mental, introduciendo otros vocabularios y marcos interpretativos, o simplemente modulando el discurso dominante desde sus propios términos? ¿Está impactando efectivamente en las prácticas profesionales, en sus marcos de interpretación y acción, o está siendo absorbido como complemento inofensivo que no cuestiona los fundamentos del saber biomédico hegemónico? 

Estas preguntas no apuntan a evaluar el éxito o fracaso de la figura desde un prisma individualista, sino a colocar a este rol profesional en un entramado de relaciones de poder y a visibilizar, desde ahí, las condiciones materiales, relacionales e institucionales que determinan si su presencia puede efectivamente abrir grietas en el orden epistémico establecido o si, por el contrario, se inscribe como mecanismo de legitimación de un sistema que incorpora formalmente la diversidad sin transformar sustancialmente sus lógicas de poder. En otras palabras, la cuestión no es tanto si los agentes de apoyo entre iguales están bien o mal integrados, sino qué tipo de transformaciones estructurales serían necesarias para que su saber pueda circular con legitimidad, interpelar los regímenes de verdad dominantes y participar activamente en la reconfiguración de lo que cuenta como conocimiento válido en salud mental. Esto exige examinar las condiciones concretas de posibilidad: ¿se les reconoce autoridad para cuestionar diagnósticos, tratamientos o intervenciones?, ¿participan en espacios de toma de decisiones o solo en funciones complementarias?, ¿sus saberes son documentados, sistematizados y transmitidos como formas legítimas de conocimiento profesional, o permanecen en el terreno de lo anecdótico o lo personal?, ¿existen mecanismos, sean institucionales o independientes, que protejan su autonomía frente a presiones para conformarse al discurso clínico hegemónico?, ¿se valoran y retribuyen sus contribuciones de manera equiparable a otros roles profesionales, o se reproducen formas de precariedad laboral que refuerzan jerarquías simbólicas?

La figura del agente de apoyo entre iguales, entonces, funciona como analizador de las tensiones irresueltas del sistema de salud mental. Su valor reside en volver visibles estas contradicciones y en instalarlas en el centro del debate sobre las condiciones necesarias para que otros saberes puedan afectar y tomar partido en el campo de la salud mental. A partir de lo expuesto, y a modo de propuesta abierta, quizás sea necesario plantear que el encuentro entre saberes profanos y saberes expertos que se produce en los contextos en donde se articula la figura del agente de apoyo entre iguales, pueda entenderse a partir de la producción necesaria de una “zona de contacto”^71^: un espacio de construcción dialógica de significados e itinerarios, una forma de escapar del corsé predefinido que suele imponer la lógica biomédica. La zona de contacto, un concepto acuñado por Mary Louise Pratt, hace referencia a ciertos espacios sociales donde culturas distintas se encuentran, chocan y se entrelazan, casi nunca en condiciones de igualdad. No es un encuentro armonioso ni neutral; es un cruce en fricción, atravesado por el conflicto, la negociación y los malentendidos, pero también por la creatividad que es necesario atravesar de forma situada y artesanal, donde el poder gravita y donde se inventan nuevas maneras de hablar, escribir y existir. Con esta noción, Pratt designa un lugar donde personas con historias, lenguas y posiciones de poder desiguales interactúan directamente. Pensar el encuentro entre saberes en términos de zona de contacto permitiría, así, profundizar y dar lugar a la necesidad de generar itinerarios de construcción compartida del conocimiento en salud mental a partir de culturas o experiencias distintas: aquellas producidas desde la experiencia vivida del sufrimiento, sus aprendizajes, acciones y silencios; y las de las disciplinas y ecosistemas epistemológicos que intentan comprenderlo, apaciguarlo, neutralizarlo o acompañarlo.

## Conclusiones

La justicia epistémica en salud mental no puede reducirse a la incorporación formal de agentes de apoyo entre iguales en los dispositivos institucionales. Como hemos argumentado, la justicia epistémica en salud mental exige una transformación radical de las condiciones estructurales que han sostenido históricamente la deslegitimación de los saberes profanos y la privación de agencia epistémica de las personas psiquiatrizadas. El agente de apoyo entre iguales, entre su potencial transformador y el riesgo de cooptación, entre la profesionalización y la pérdida de su dimensión política, y entre el reconocimiento formal y la ausencia de cambios estructurales en la producción y validación del saber en salud mental exige la creación de zonas de contacto desde donde trabajar colaborativamente en la producción de significados e itinerarios. Su valor analítico reside en su capacidad para evidenciar que la transformación del sistema no parece ser una cuestión técnica o de incorporación de nuevos roles, sino política y organizativa, que articule el reconocimiento y el fortalecimiento de las fraternidades epistémicas y los movimientos en primera persona como activos comunitarios^72^, como espacios colectivos de construcción de saber; la transformación profunda de las culturas profesionales y de formación en salud mental, incorporando perspectivas que integren saberes profanos y marcos de derechos humanos; la disputa de los marcos regulatorios, las políticas públicas y los modelos de financiación que estructuran el campo; y la articulación de procesos de coproducción local de sentido que reconozcan los activos comunitarios como fundamento de intervenciones territorialmente situadas. 

La justicia epistémica, entonces, no se alcanza mediante la inclusión formal de sujetos previamente excluidos en espacios que mantienen intactas sus lógicas de funcionamiento, sino mediante la transformación radical de las condiciones estructurales, materiales y simbólicas que hicieron posible su exclusión. Es desde este reconocimiento desde donde se vuelve posible habilitar formas de cuidado que reconozcan plenamente a quienes reclaman hoy el derecho legítimo a nombrar su propia experiencia, a construir colectivamente marcos interpretativos alternativos y a participar activamente en la definición de otros horizontes posibles para la salud mental. Cabría preguntarse: ¿puede existir un sistema de salud mental que no necesite incorporar la diversidad epistémica para legitimarse, sino que esté construido desde el principio sobre la pluralidad de formas de saber sobre el sufrimiento humano como condición de su propia existencia, de su propio orden?
