# Supplementary material for: Saberes profanos y justicia epistémica en salud mental: análisis crítico del agente de apoyo entre iguales en el Estado español
Source: Salud Colect. 2026 Feb 26;22:e6041. [Article in Spanish] doi: 10.18294/sc.2026.6041 (PMC13448053; doi:10.18294/sc.2026.6041)
Supplement: Translation

# Translation: Lay knowledge and epistemic justice in mental health: a critical analysis of the peer support worker in the Spanish State

**Authors:** Diana Gonzalez-Mañas, Martín Correa-Urquiza

**Affiliations:** 1 Corresponding author. Master’s Degree in Medical Anthropology and Global Health. Research Assistant, Universidad de Barcelona, Barcelona, Spain. dianagonzalez@ub.edu; 2 Doctor of Medical Anthropology and International Health. Professor Serra Hunter, Department of Anthropology, Philosophy, and Social Work. Universidad Rovira i Virgili, Tarragona, Spainmartin.correaurquizav@urv.cat

**Keywords:** Mental Health, Epistemology, Medical Anthropology, Human Rights, Spain

## Abstract

This essay critically examines the figure of the peer support worker within the mental health system of the Spanish State as a dispositif situated in the tension between the transformation of epistemic hierarchies and the risk of institutional co-optation. Drawing on our experiences and research in the field of critical medical anthropology and human rights in mental health, together with and from the activisms of the Mad Movement in the Spanish State, we analyse how this professional figure simultaneously embodies a promise of epistemic power redistribution and the danger of neutralisation by the hegemonic biomedical model. We argue that epistemic justice in mental health requires the radical transformation of the material, relational, and institutional conditions that have historically sustained the delegitimation of experiential knowledge. Through a genealogical analysis of the peer support worker and its processes of institutionalisation, we identify the structural conditions that determine whether its incorporation can effectively challenge dominant regimes of truth or, conversely, operates as a mechanism of legitimation that formally incorporates diversity without transforming existing logics of power. We conclude by proposing a framework of four dimensions of political intervention: strengthening epistemic fraternities and Mad Movement networks as community assets, transforming professional cultures, contesting regulatory frameworks, and articulating local processes of knowledge co-production.

## Introduction

Historically, the field of mental health has constituted a domain marked by profound epistemic inequalities. Although the psychiatric field has long contained internal disputes, critical currents, and counter-hegemonic experiences,^1^^,^^2^^,^^3^ the dominant biomedical orientation has tended to establish asymmetrical relations with the knowledge derived from the lived experience of psychiatrised people. Since the emergence of modern psychiatry, some positions and initiatives have questioned biomedical reductionism and the silencing of lived experience.^4^^,^^5^^,^^6^ However, these currents have largely occupied interstitial positions within psychiatric power and have not displaced the hegemony of the biomedical paradigm in institutions, public policies, or professional training. Instead, the field has privileged interpretive frameworks centred on diagnosis, pathologisation, and intervention over the recognition of psychiatrised people as subjects capable of producing valid knowledge.^7^^,^^8^^,^^9^^,^^10^

This epistemological order has configured psychiatrised people as subaltern subjects deprived of epistemic agency^11^ and, in the terms of Huertas,^12^ stripped of words. In light of the work of Miranda Fricker,^13^ this form of delegitimisation is known as epistemic injustice: a distinctive type of harm that affects individuals in their capacity as knowers. Epistemic injustice manifests paradigmatically in contexts where certain individuals or collectives face systematic discredit as bearers of valid knowledge due to structural prejudices operating through their social identity.

In the specific context of psychiatrised people, this injustice appears both in its testimonial dimension (when others receive their accounts with a credibility deficit or directly invalidate them) and in its hermeneutical dimension (when they lack the interpretive resources necessary to articulate and render their experience intelligible within a sociocultural context that has marginalised their voices from the processes that construct legitimate language).^11^^,^^13^^,^^14^ Nevertheless, in recent decades an epistemological shift has begun to challenge this hierarchical distribution of knowledge. The emergence of what has been conceptualised as lay knowledge^10^ has disrupted this order as both a political and epistemic response to historical silencing. This development has opened a horizon that seeks to repair the forms of epistemic injustice that, following Fricker, have systematically harmed psychiatrised people, both in their condition as knowers and in their capacity to articulate and interpret their own experience.

In the specific context of Spain, the gradual expansion and consolidation of the Mad Movement or first-person movement has highlighted the need to reconsider the asymmetrical distribution of legitimacy in the field of epistemic production.^15^ These initiatives have explicitly and forcefully problematised the fact that, for centuries, those who have directly experienced madness have faced structural exclusion from institutional spaces of discursive production.^16^ Within this interpretive framework, the notion of speaking “in the first person” conceptualises the singular and plural dimensions of individuals, associations, collectives, and organisations developed by people with psychic suffering who have lived through experiences of psychiatrizsation. This notion does not constitute a closed category and remains subject to tensions, yet actors currently use it to refer to what emerges from individuals and collectives themselves. Ultimately, it has constituted a political act: abandoning the condition of a passive object of others’ discourse in order to claim the legitimate authority to interpret, to name, and to construct alternative paradigms.^15^^,^^17^^,^^18^^,^^19^

In this context, lay knowledge constitutes a body of knowledge that emerges directly from the lived experience of affliction. It does not concern suffering alone; rather, it provides the very foundation from which individuals configure the meaning of lived experience and articulate possible strategies for greater well-being. In this sense, lay knowledge represents a transformative praxis oriented toward generating material and symbolic conditions for horizontal dialogue in which psychiatrised people finally gain recognition as subjects who produce meaning rather than merely objects of intervention or knowledge.

The emergence of lay knowledge, however, does not occur on epistemologically neutral ground. From the critical perspective of medical anthropology, Arthur Kleinman^20^ analyses the distinction between different epistemological frameworks in the field of health and develops a theoretical differentiation among the concepts of disease, illness, and sickness. For Kleinman, the subjective experience of suffering (illness) and the sociocultural forms through which societies interpret it (sickness) often become invisible within the hegemonic biomedical narrative, which focuses exclusively on the objectified conceptualisation of disease (disease). Building on this theoretical line, Martínez-Hernáez and Correa-Urquiza^15^ propose conceptualising lay knowledge as nomadic knowledge that positions itself laterally in relation to the institutionalised expert apparatus. Such knowledge does not traditionally originate within a formalised academic discipline and does not articulate itself through a logic of authority. Instead, it emerges within the liminal interstice between embodied experience and existential necessity. Its essentially nomadic character makes it resistant to institutional codification and, precisely because of this feature, potentially disruptive to the established epistemic order. What stands fundamentally at stake in the field of lay knowledge, therefore, does not simply involve formally recognising an “other” subject; rather, it involves radically transforming the structural conditions under which knowledge production takes place in the field of mental health and dismantling the hegemonic narrative that has historically defined lived experience from an objectifying external standpoint.

Drawing on our experiences and research in medical anthropology and human rights in mental health, together with and from the activisms of the Mad Movement in Spain, this article critically examines the possibilities, tensions, and conditions under which the peer support worker may contribute to an effective redistribution of epistemic authority and legitimacy within the mental health system. It also explores the scenarios in which this figure risks being absorbed, neutralised, or instrumentalised by the logics of the expert system.

In this sense, when we refer to the mental health system, we mean the network of cultural, economic, and institutional dimensions that operate from care services to regulatory frameworks, modes of financing, the design of public policies, intervention protocols, and the professional cultures that shape these services and resources. Although different epistemological orientations, intervention paradigms, and initiatives promote the transformation of mental health systems toward human rights-based models,^21^^,^^22^^,^^23^ this heterogeneity does not prevent biomedical-psychiatric knowledge from occupying a hegemonic position in defining what counts as pathological and, consequently, a hegemonic position in defining recovery.^24^ This tension marks precisely the terrain where the peer support worker can reveal the conditions that make radical transformation possible.

This essay develops around three main axes. First, we examine the figure of the peer support worker as a device situated between different forms of knowledge, focusing on its genealogy and its potential to reconfigure epistemic and power relations. Second, we critically analyse the risks of institutional co-optation that may neutralise its transformative capacity when the peer support worker becomes integrated under professional logics that reproduce the hierarchies it originally challenged. Finally, we propose a framework of structural conditions necessary to advance toward epistemic justice in mental health, understood not as a state achievable through the formal incorporation of the peer support worker but as a political horizon that requires a radical transformation of the conditions under which knowledge is produced and legitimised. Throughout this discussion, the peer support worker does not appear as a solution in itself for transforming the mental health system; rather, it functions as a privileged analytical lens that allows us to interrogate both the conditions that make such transformation possible and its structural limits. Ultimately, the peer support worker does not naturally resolve the problem of epistemic injustice, but it makes that problem visible and establishes it as an unavoidable political issue.

## Genealogy and potential of the peer support worker

The professional role of the peer support worker constitutes a critical innovation in the field of mental health that largely responds to the complex processes through which psychic suffering has become politicised and through which lay knowledge has gained legitimacy, processes that have expanded significantly over the past decades.^25^^,^^26^ Although early experiences of peer support in mental health already appear in the eighteenth century,^27^ its roots gained strength through the user and psychiatric survivor movements that emerged during the 1960s and 1970s, particularly in the United States and Western Europe.^28^^,^^29^ These movements developed as a critical response to institutionalisation practices, coercive interventions, and the systematic exclusion of psychiatrised people from decision-making spaces concerning their own lives.^30^ Pioneering organisations such as the Mental Patients’ Liberation Front in Boston and the Campaign Against Psychiatric Oppression in the United Kingdom articulated forms of horizontal support that challenged the professional monopoly over care and asserted lived experience as a legitimate source of knowledge and authority.^31^

Within this context, in the early 1980s Gartner and Riessman^32^ described mutual support in mental health as a process of aid among people who share similar experiences or difficulties, oriented toward both personal change and social transformation. Later, at the beginning of the twenty-first century, Mead, Hilton, and Curtis^33^ expanded this conceptualisation by defining peer support as a reciprocal relationship of exchange grounded in respect, shared responsibility, and the collective construction of what proves useful for those who participate in that relationship. In short, peer support functions as a political practice that resists the power relations inherent in biomedical-psychiatric knowledge and promotes forms of mutuality based on a shared understanding of suffering.^33^^,^^34^^,^^35^

From the 1990s onward, with the emergence of the recovery model,^36^ peer support began to undergo processes of formalisation and progressive institutionalisation. It gradually became integrated - at different levels - into the mental health systems of numerous countries,^33^^,^^37^^,^^38^ generating debates and controversies regarding the most appropriate way to incorporate into services a force that actors originally conceived as alternative and critical.^39^^,^^40^^,^^41^^,^^42^^,^^43^ In its ideal epistemological and ontological formulation, the peer support worker embodies a radical attempt to fracture the traditional monopoly of expert knowledge and to recognise lived experience as a legitimate source of knowledge. This professional role promotes the development of innovative forms of support characterised by horizontality and greater openness to the irreducible singularity of subjective life trajectories.^28^^,^^44^^,^^45^ Its differential epistemic positioning lies in the fact that it constructs knowledge from embodied experience, practices listening from the existential immediacy of “having been there,” and develops forms of accompaniment grounded in experiential proximity.^46^^,^^47^

In certain institutional contexts, the peer support worker primarily accompanies people through processes of personal recovery; in others, this professional role facilitates mutual support groups or mediates between service users and health professionals.^33^^,^^48^ In Spain, the incorporation of the peer support worker has developed unevenly and remains incipient, with significant yet fragmented advances across autonomous communities.^26^ Most initiatives originate in associations and third-sector organisations, while integration into the public system remains limited and lacks a homogeneous professional category.

Among the most significant potentialities of this interstitial role lies its capacity to establish and sustain horizontal care relationships grounded in practices of embodied listening, situated empathy, and mutual recognition.^49^ One of the aspects most valued by people accompanied by peer support workers involves precisely the possibility of constructing an identity not reduced to a pathological condition, recovering agency over one’s own life narrative, and envisioning alternative futures beyond the determinism of psychiatric prognosis.^50^ Moreover, this professional role may contribute to redistributing the power relations embedded within care institutions. The process of deinstitutionalisation in Trieste (Italy), for example, unfolded alongside the incorporation of psychiatrised people as workers in community services across the region. This development occurred simultaneously with a radical critique of asylum-based logics, an effective democratisation of care relationships, and an explicit politicisation of institutional practices.^51^^,^^52^ In that context, the articulation between institutional transformation and the recognition of lay knowledge demonstrated its effectiveness both clinically and socially, turning the Trieste model into an international reference for the democratisation of the mental health field.

## Peer drift and institutional neutralisation

Nevertheless, the transformative potential of the peer support worker should not lead to an idealised or uncritical conception of this role, nor should it obscure the multiple structural risks surrounding its process of institutionalisation. One of the principal challenges identified in the literature emerges when its transformative potential coexists dialectically with a persistent risk of institutional neutralisation and systemic co-optation.^53^ In such cases, the peer support worker may gradually become an institutionally sanitised version of the “good patient,” rather than maintaining its potential as a critical, disruptive, and collectively articulated role. When institutions assign instrumental functions to the peer support worker without granting effective epistemic recognition, require them to reproduce preexisting logics uncritically, or redirect their responsibilities away from the distinctive practices that differentiate them from other professionals, their disruptive capacity can become severely limited.^54^

Scholars refer to this phenomenon as peer drift, and the literature identifies it as one of the central challenges to integrating peer support within mental health services.^55^^,^^56^ In particular, it raises the question of whether peer support maintains its original essence once it moves from everyday and community environments into the structured and regulated context of mental health systems. More specifically, institutionalisation prompts a key question: does peer support remain the same form of support in terms of authenticity, or do its meaning and function transform once it operates within these systems?^57^

A paradigmatic manifestation of this neutralising drift appears in the institutional role of the “expert patient”.^10^ Although this role does not constitute a peer support initiative as such, it also originates from the fact of having lived through a specific mental health experience. In these cases, the health system formally recognises the voice of experience only when that voice subordinates itself to its pre-established interpretive frameworks: when the subject designated as “expert” reproduces the hegemonic biomedical discourse uncritically, actively promotes psychoeducation and adherence to pharmacological treatment, or performs intermediary functions between health professionals and service users. Evidence also documents that some individuals who assume this institutional role end up experiencing feelings of isolation, lack of protection, or instrumentalisation by organisational structures that do not adequately recognise either their epistemic specificity or their distinctive contribution.^58^^,^^59^^,^^60^

Within care systems, scholars have identified several structural obstacles. These include difficulties achieving effective integration into multidisciplinary teams; the persistence of skeptical or openly hostile attitudes among professionals trained in traditional biomedical models; ambiguities and conflicts in defining roles and responsibilities; the absence of clear protocols regarding confidentiality and ethical boundaries; and rigid organisational cultures that make it difficult to adapt to forms of work grounded in horizontality and mutuality.^61^^,^^62^

In short, the hybrid character of the peer support worker places this professional role under the constant risk that institutions will instrumentalise these forms of knowledge and recognise them selectively only insofar as they remain subordinate to the dominant biomedical logic. However, the most profound critiques emerge precisely from first-person movements themselves, which problematise the very professionalisation of peer support as a potential betrayal of its emancipatory foundations. Their concern centers on the transformation of the peer support worker into an “expert” or a bearer of “technical knowledge” who might paradoxically reproduce the same epistemic and relational hierarchies that the movement originally sought to challenge. In such cases, rather than transforming the system, the individual ends up adapting to its norms, values, and modes of operation.^26^

Observers also warn that excessive anchoring in the identity of a service user may hinder professional integration and limit the capacity to exert influence within teams.^26^ Under these conditions, instead of opening spaces for genuine epistemic recognition, the incorporation of the peer support worker into the mental health system may close off the conflict inherent to the coexistence of diverse forms of knowledge, reduce lay knowledge instrumentally to a subordinate technical function, and neutralise its transformative potential.^63^

Part of this dynamic resonates with the critique articulated by the Indian philosopher Gayatri Chakravorty Spivak, who argues that the issue does not lie in the subaltern’s inability to speak (understood as a positional condition rather than an identity), but rather in the socially limited possibilities for their speech to be heard and resonate.^64^ For Spivak, the subaltern fails in the attempt to communicate because interpretive frameworks encapsulate their message within a mode that individualises the enunciation and links it to personal problems - here, problems supposedly related to psychic suffering. In other words, the issue does not concern an absence of voice or muteness, but rather the closed interpretations imposed on the act or message that prevent the information from being received and recognised.

To this discussion we must add the need to consider whether, beyond the existing training programs in peer support,^26^^,^^65^^,^^66^ this professional role should also emerge from processes of shared meaning-making, from a diverse body-text, minimally consensual, that arises from organised social movements. Fernando Broncano^67^ argues that the lived experience of oppression and subalternisation does not suffice, on its own, to develop a critical awareness of one’s situation; such awareness requires encounter and problematisation with a similar other. For Broncano, lay knowledge does not end with individual experience; it requires epistemic communities in which lived experience can articulate itself as collective and political knowledge. In this sense, the role of the peer support worker as an epistemic fraternity^18^^,^^67^ may prove fundamental for an integration that reflects the emancipatory power of the movements from which peer support originally emerged.

For this reason, it also becomes essential that peer support workers maintain and develop connections with social movements linked to the Mad Movement, as these movements constitute privileged sites for producing critical consciousness and generating collective epistemic knowledge. If mental health systems incorporate peer support workers, perhaps we should recognise that they represent a form of collective knowledge constructed in community - an epistemic fraternity - whose inclusion in care teams must preserve the transformative and liberatory spirit of the social movements from which they emerged.

Regarding lay knowledge, defining it exclusively as knowledge derived from experience may be reductive. Undoubtedly, it also involves knowledge that does not limit itself to experience alone; rather, it concerns knowledge about - or in relation to - the existential dimension of suffering, a dimension that symptom-based disciplines frequently overlook. Lay knowledge is not merely a personal story. It also constitutes a form of wisdom about the human condition, about how existential suffering manifests and how people live through it. This philosophical-existential knowledge, which people with experiences of psychiatrisation may develop, complements and enriches the biomedical model centered on symptoms.

In a recent interview,^68^ the Dutch psychiatrist Jim van Os recalls that mental ill-health includes an existential dimension that the current model of care has systematically disregarded. The reductionism that frequently characterises the field of mental health has led us to the fallacy of believing that these forms of suffering consist only of pathological realities manifested as symptoms, and that diagnoses constitute natural entities located in individuals’ brains. The existential dimension - often eclipsed by symptom-based languages - constitutes precisely one of the epistemic cores from which lay knowledge emerges. It opens the possibility of understanding mental distress in more complex ways, ways that do not reduce lived experience to a set of symptoms.

## From formal incorporation to structural transformation: conditions for epistemic justice

In light of Miranda Fricker’s work, epistemic justice does not appear as a state that can be definitively achieved; rather, it functions as an ethical and political horizon that demands recognition of other forms of knowledge, the dismantling of epistemic hierarchies, and the creation of institutional and community contexts that enable genuine dialogue among experiences, disciplines, and ways of understanding suffering.^69^ In this sense, the peer support worker may be understood as a liminal role situated at the threshold between historically hierarchical forms of knowledge. It may function simultaneously as promise and risk, as bridge and tension, as an opportunity to reconfigure relations among forms of knowledge, provided that the logics of professionalisation serving the expert system do not neutralise its critical potential.

Since the role of the peer support worker has revealed itself as a liminal position,^70^ its emergence may be interpreted both as a sign that expert knowledge has begun to open itself to subaltern forms of knowledge and, paradoxically, as an attempt to contain and redirect that emergence toward logics that remain functional to existing frameworks. For this reason, reflection must move beyond abstract assessments of this role’s potential and toward concrete questions about its practical configuration within specific institutional contexts. What kinds of tensions actually shape the everyday practice of the peer support worker when their knowledge and positionalities encounter clinical apparatuses? Do genuine spaces for deliberation and horizontal knowledge construction emerge? To what extent can this professional role facilitate a shift from expert knowledge toward embodied forms of knowing? Does it expand the boundaries of what can be said in mental health by introducing new vocabularies and interpretive frameworks, or does it merely modulate the dominant discourse in its own terms? Does it effectively transform professional practices, their interpretive frameworks, and their modes of action, or does the system absorb it as a harmless complement that leaves the foundations of hegemonic biomedical knowledge intact?

These questions do not seek to evaluate the success or failure of this figure from an individualistic perspective. Rather, they situate this professional role within a network of power relations and, from that vantage point, make visible the material, relational, and institutional conditions that determine whether its presence can effectively open cracks in the established epistemic order or, on the contrary, function as a mechanism that legitimises a system that formally incorporates diversity without substantially transforming its logics of power. In other words, the central issue does not concern whether peer support workers are well or poorly integrated, but rather what kinds of structural transformations would allow their knowledge to circulate with legitimacy, challenge dominant regimes of truth, and actively participate in redefining what counts as valid knowledge in mental health.

Addressing this issue requires examining concrete conditions of possibility: Do institutions recognise their authority to question diagnoses, treatments, or interventions? Do they participate in decision-making spaces, or do institutions confine them to complementary functions? Do professionals document, systematise, and transmit their knowledge as legitimate forms of professional knowledge, or does it remain confined to the realm of the anecdotal or the personal? Do institutional or independent mechanisms exist that protect their autonomy from pressures to conform to hegemonic clinical discourse? Do organisations value and remunerate their contributions in ways comparable to other professional roles, or do they reproduce forms of labor precarity that reinforce symbolic hierarchies?

The professional role of the peer support worker, therefore, functions as an analyser of the unresolved tensions within the mental health system. Its value lies in making these contradictions visible and placing them at the center of debates about the conditions necessary for other forms of knowledge to affect - and take part in - the field of mental health. Based on the arguments developed above, and as an open proposal, it may be useful to conceptualise the encounter between lay knowledge and expert knowledge that emerges in contexts where the peer support worker operates as the production of a “contact zone”:^71^ a dialogical space for constructing meanings and trajectories, and a way to escape the predefined corset that biomedical logic often imposes.

The contact zone, a concept coined by Mary Louise Pratt, refers to social spaces where distinct cultures meet, clash, and intertwine, almost never under conditions of equality. It does not describe a harmonious or neutral encounter; rather, it designates a site of friction shaped by conflict, negotiation, and misunderstanding, but also by the creativity that must be navigated in situated and often artisanal ways - spaces where power gravitates and where new ways of speaking, writing, and existing emerge. Through this notion, Pratt identifies a place where people with different histories, languages, and unequal positions of power interact directly. Thinking about the encounter between forms of knowledge in terms of a contact zone would therefore allow us to deepen the analysis and foreground the need to generate shared pathways for constructing knowledge in mental health across different cultures and experiences: those produced through the lived experience of suffering - its learnings, actions, and silences - and those emerging from the disciplines and epistemic ecosystems that seek to understand, soothe, neutralise, or accompany it.

## Conclusions

Epistemic justice in mental health cannot be reduced to the formal incorporation of peer support workers into institutional settings. As we have argued, achieving epistemic justice in mental health requires a radical transformation of the structural conditions that have historically sustained the delegitimisation of lay knowledge and the deprivation of epistemic agency among psychiatrised people. The peer support worker stands at the intersection of multiple tensions: between transformative potential and the risk of co-optation; between professionalisation and the loss of its political dimension; and between formal recognition and the absence of structural changes in the production and validation of knowledge in mental health. These tensions demand the creation of contact zones from which actors can work collaboratively in the production of meanings and trajectories.

The analytical value of this figure lies in its capacity to reveal that transforming the mental health system does not appear to be a merely technical matter or one that can be resolved by introducing new professional roles. Rather, it constitutes a political and organisational challenge. Addressing it requires articulating several processes: recognising and strengthening epistemic fraternities and first-person movements as community assets^72^ and as collective spaces for the production of knowledge; promoting a profound transformation of professional cultures and educational frameworks in mental health by incorporating perspectives that integrate lay knowledge and human rights approaches; contesting the regulatory frameworks, public policies, and funding models that structure the field; and developing local processes of the co-production of meaning that recognise community assets as the foundation of territorially situated interventions.

Epistemic justice, therefore, does not arise through the formal inclusion of previously excluded subjects within spaces that leave their logics of operation intact. Instead, it requires a radical transformation of the structural, material, and symbolic conditions that made their exclusion possible. Only through this recognition can new forms of care emerge - forms that fully acknowledge those who today claim the legitimate right to name their own experience, to collectively construct alternative interpretive frameworks, and to participate actively in defining new horizons for mental health.

This reflection invites a final question: Can a mental health system exist that does not need to incorporate epistemic diversity in order to legitimise itself, but is instead built from the outset upon the plurality of ways of knowing human suffering as a condition of its very existence and order?
